# The evolution of genetic covariance and modularity as a result of multigenerational environmental fluctuation

**DOI:** 10.1093/evlett/qrad048

**Published:** 2023-10-17

**Authors:** Isabela do O, Michael C Whitlock

**Affiliations:** Department of Ecology and Evolution, University of Lausanne, 1015 Vaud, Switzerland; Zoology Department, University of British Columbia, Vancouver, BC V6T1Z4, Canada; Zoology Department, University of British Columbia, Vancouver, BC V6T1Z4, Canada

**Keywords:** pleiotropy, genetic covariance, modularity, **G**-matrix, changing environments

## Abstract

The genetic covariance between traits can affect the evolution of a population through selection, drift, and migration. Conversely, research has demonstrated the reciprocal effect of evolutionary processes on changing genetic covariances, in part through mutational covariance, correlational selection, and plasticity. In this article, we propose that correlated changes in selective optima over generations can cause the evolution of genetic covariance and the **G**-matrix in such a way that the population can, in the future, evolve faster. We use individual-based simulations of populations exposed to three types of changing environments that differ in the correlation of the change between selective pressures. Our simulation experiments demonstrate that selection pressures for different traits changing in a correlated pattern over generations can lead to stronger trait correlations compared to the case with independently changing selective optima. Our findings show that correlated selective pressures result in significantly higher genetic trait covariance and that pleiotropy accounts for the majority of the difference in covariance between treatments. We also observe that the mutational variance evolves according to the environment that the populations were exposed to. Moreover, we show that clustered patterns of changes in selection can allow the evolution of genetic modularity. We show that the pattern of change in the selective environment affects the pace at which fitness evolves, with populations experiencing correlated change in optima having on average higher mean fitness than those experiencing uncorrelated environment change.

## Introduction

Multiple traits are often affected by the same genes or genotypes, similar environments, and overlapping developmental processes, which generates covariation of these traits among individuals. The pleiotropy caused by shared genetic and developmental bases of trait development is a common feature of developmental systems, and this pleiotropy allows for genetic covariance of traits. (Besides pleiotropy, the nonrandom association of alleles at different loci, linkage disequilibrium, also can cause genetic covariance of traits.)

Genetic covariance can impact the evolutionary path of a population, as selection on one trait would cause indirect correlated responses in other traits (Arnold et al., 2008; Lande, 1979; Svensson et al., 2021; Turelli, 1988; Walsh & Lynch, 2018; Wood & Brodie, 2015). Genetic correlations can either increase or decrease responses to selection; when favored traits are positively correlated, response is accelerated; when they are negatively correlated, response is hindered (Walsh & Blows, 2009). A long-standing question in quantitative genetics is whether such genetic correlations meaningfully limit or enhance the evolution of populations over time (Agrawal & Stinchcombe, 2009). The significance of these constraints over large evolutionary time scales is still being uncovered (Rohner & Berger, 2023).

Schluter (1996) showed that the direction in phenotypic space of divergence between taxa is biased towards the major axes of genetic variation in multidimensional trait space (in other words, the first principle component of the additive genetic variance–covariance matrix, **G**). Schulter’s (1996) findings have been repeatedly observed in the decades since, especially in the ground-breaking work of Houle et al. (2017). The bias in divergence is predicted by a model that assumes that the trajectory of evolution is constrained by available genetic variation. However, such a connection between patterns of genetic variation and divergence may also be expected because of causality in the opposite direction—selection may directly or indirectly affect the pattern of genetic variability in ways that bias the pattern of genetic variation by the direction of selection causing divergence (Houle et al., 2017; Schluter, 1996). In this article, we will explore a new mechanism for such effects caused by multigenerational correlated changes in selected optima.

Several possible adaptive explanations for the evolution of covariance have been described. Multiple authors (e.g., Roff & Fairbairn, 2012; Sinervo & Svensson, 2002; Svensson et al., 2021) have shown that correlational selection can cause the evolution of genetic correlations. Correlational selection occurs when the fitness of certain combinations of traits defines a ridge on the adaptive landscape, that is, when the optima of one trait depends linearly on the value of another trait in the same individual (Svensson et al., 2021). Jones et al. (2014) described a changing mutation model where the distribution of pleiotropic effects created by mutation is fixed, so the changes in genetic covariance they observe are caused by the selective sorting of those mutations. In addition, a developmental system that creates well-matched combinations of traits will be, on average, more fit than a development that does not bias toward fit combinations. As a result, correlational selection selects for pleiotropic genetic effects that, in turn, create positive covariance in the population.

The work we refer to here, as well as in our results below, considers a positive correlation between the selection on the traits. In these circumstances, a positive covariance in the trait values increases the evolvability of the organisms, and this is the pattern observed. In environments with favored negative correlational selection, these results would predict, by symmetry, negative genetic covariance. For the sake of simplicity in this article, we only consider the positive correlation of the selection, and therefore we refer to covariance in the traits along the same axis as “positive covariance.”

Another potential selective cause of genetic correlations is correlated optima of traits across local populations in a spatially structured metapopulation (Guillaume & Whitlock, 2007). Local selection tends to take local populations toward local optima, and if those local optima have correlated values for multiple traits, genetic correlations in those traits will be observed over the metapopulation. Gene flow between diverging populations then introduces local genetic variation in the direction of the selective divergence among populations.

Plastic responses to varying selective pressures may also lead to the evolution of genetic correlations. Phenotypic plasticity requires flexible developmental systems, such that development is capable of changing in phenotypic directions favored by the optimal plastic response to varying environments. Draghi and Whitlock (2012) have shown that the evolution of phenotypic plasticity in response to temporally heterogeneous selection with correlated optima of traits leads to the evolution of greater pleiotropy, stronger genetic correlations, and correlated patterns of mutational effects.

Finally, continuous unidirectional movement of the optimum in multidimensional space promotes stronger covariance (Chebib & Guillaume, 2022; Jones et al., 2004; Pavlicev et al., 2011). The continuous unidirectional movement of a single peaked fitness optima exposes the population to directional selection in multiple-dimensional space (Melo & Marroig, 2015; Pavlicev et al., 2011). However, it is unclear how many traits experience sustained directional selection in the long term, so it is unclear how biologically relevant such a process may be to how many traits. One ecologically relevant pattern that may sustain directional selection on traits over indefinite evolutionary time is temporally fluctuating selection (Pavilicev et al., 2011), which we consider here.

Patterns of the genetic relationships among traits extend deeper than what is captured in the genetic covariance alone; clusters of traits sometimes covary together, a pattern called “variational modularity” (Melo et al., 2016). Modularity has been suggested to evolve as an effect of a changing environment (Wagner & Altenberg, 1996). Developmental systems are modular when the molecules or structures involved in some traits interact within clusters, a process that involves pleiotropy. Such pleiotropy leads to variational modularity (Melo et al., 2016).

In a changing environment, the modularity of the gene-to-phenotype map can become more important to the pace of evolution. Lipson et al. (2002) have pointed out that a changing environment should lead to the spontaneous evolution of modularity. Clune et al. (2013) and previous authors (Kashtan & Alon, 2005; Kashtan et al., 2007) predict that modularity will emerge in a rapidly changing environment with similar pressures on different subsets of traits. While other work has shown the emergence of modularity as a result of changing environments (He et al., 2009; Kashtan & Alon, 2005; Kashtan et al., 2007), here we investigate it using quantitative traits and population-level quantities, such as genetic covariance. In this article, we investigate the evolution of variational modularity at a population level through the evolution of genetic covariances.

In this article, we explore a new context for the evolution of pleiotropy and genetic correlations of traits. Here we explore how fluctuating and correlated changes in the selective environment over generations can affect the evolution of pleiotropy and genetic covariances. Organisms live in changing environments as a result of both predictable daily, seasonal, or geological cycles, and stochastic changes such as natural disasters and human intervention (Mbogo et al., 2003; McPhillips et al., 2018; Menge & Sutherland, 1976; Yuan et al., 2017). These environmental changes offer potential for evolution on a short time scale (Chesson & Huntly, 1997; Foflonker et al., 2015; Wolff, 1996), likely affecting multiple traits at once. Moreover, disturbances in one aspect of the environment may be linked to others, as seen in the frequent association between rising ocean temperature and water acidification (Doney et al., 2012; Laubenstein et al., 2018). These co-occurring disturbances lead to interacting effects on the selective pressure of different traits (Domenici et al., 2014; Kroeker et al., 2013; Riebesell & Gattuso, 2015), where a change in one parameter (e.g., water temperature) can intensify the sensitivity to selection in another parameter (e.g., pH) (Kroeker et al., 2013).

Previous research has shown that temporally heterogeneous selection may promote the development of pleiotropy via the evolution of plasticity (Draghi & Whitlock, 2012), but that work did not consider evolution in the absence of plasticity or over multigenerational shifts in adaptive optima. Correlated changes in the fitness optima of multiple traits over generations allow a recurrent mechanism for continued directional selection (Pavlicev et al., 2011). Our hypothesis is that environments that change selective optima in correlated ways should create the sustained correlated directional selection that can allow the evolution of genetic correlations in adaptive directions and a faster evolution of population mean fitness.

Our main hypothesis is that when environmental change exposes multiple traits to variation in selective pressure that follows correlated patterns, pleiotropy and genetic covariance between these traits will evolve. To test this hypothesis, we run a series of simulations of evolving populations subject to selection on some traits with explicit and evolving genetic bases. We simulate populations exposed to trait optima that change over generations. The core experiments in the article compare a situation in which the trait optima changes in a correlated way over time (“correlated changing environment”) to cases where the optima of different traits change independently (“independently changing environment”). We also analyze the evolution of mutational variance and covariance, showing that the evolved pleiotropy in these correlated environments leads to correlations in the effects of new mutations. Our main goal is to observe and contrast the evolution of total genetic covariance—and the rate of evolution of fitness that results—in these different simulated environments.

Moreover, we examine the effect of “block-correlated” changes in optima, that is, when traits from the same “block” (non-overlapping subset of traits) covary over time in their optima, and the optima of traits from different blocks change in an uncorrelated way. This final series of experiments looks at the evolution of modularity among traits due to changes in their optima over time. Our results will show that the multidimensional pattern of environmental change over time can strongly influence the evolution of pleiotropy, genetic covariance, and trait modularity.

## Methods

### General parameters

Our model considers a haploid population with a constant number of individuals, *N*. We characterize individuals by their genotype, which has two types of heritable components. One type of gene controls the magnitude of the effect on phenotypes. We call these the “weight” genes, and the *j*th weight gene has an effect *a*_*j*_, where *a*_*j*_ ∈ ℝ. The other type of gene defines which weight gene contributes to which trait. We represent this portion of the genotype as a matrix, called the contribution matrix, and the entries we refer to as *c*_*ij*_, which describes the effect of weight gene *j* on trait *i*. Each weight gene and each entry of the ***c***-matrix represents an independent gene, *c* ∈ ℝ^2^. For our default case with four traits and four weight genes, there are 20 genes in the genome.

Individuals’ phenotypes depend on their genotypes. To calculate the phenotype, the product of the weight genes and their corresponding contributions are summed. The phenotypes *z* can be represented by a vector, where *z*_*i*_ ∈ ℝ:


$$\begin{array}{l} z_{i}=\overset{n}{\underset{j=1}{\sum }}c_{ij}a_{j} \end{array}$$

The fitness of an individual depends only on its phenotype. We model a Gaussian fitness landscape with optima varying over time and variance of one. For a given environment with phenotypic optimum *z*_*o*_, fitness is calculated as:


$$\begin{array}{l} w(z)=exp\left[-\overset{n}{\underset{i=1}{\sum }}\frac{{\left(z_{i}-z_{io}\right)}^{2}}{2}\right] \end{array}$$

Note that in all models we consider, fitness is determined multiplicatively across traits, with no correlational selection within generations.

Our model was implemented in Python version 3.6.2 using module NumPy (Harris et al., 2020) and pandas (McKinney et al., 2010). We used a separate script also in Python for the statistical tests. The source code and the entire data related to this article is available at https://github.com/isadoo/doO_Whitlock2023.

### Birth–death process

We use a Moran birth–death model, holding the population size constant over time at 1,000 individuals. At each time point, two main events happen: The birth of a new individual and the death of another. The individual that gives birth is randomly drawn from a distribution where each individual's probability of producing an offspring is proportional to its fitness. The new offspring will replace another individual that dies; which individual dies is chosen randomly with equal probability for each individual.

When a birth happens, mutations may occur on either the weight vector or the contribution matrix for the new individual. A mutation happens in each component of the weight vector with probability of μ_a_. There is a probability of μ_c_ for each of the contribution genes to have a mutation. The effect of the mutation is chosen from a normal distribution centered at 0 with a variance of 0.5; this is the case for both mutations on the weight vector and on the contribution matrix. The mutated alleles’ value is the sum of the parental value and the change due to the new mutation.

Our populations can be either asexual or sexual. With sexual reproduction, two individuals are chosen based on fitness to give birth. The offspring have a combination of the chosen individuals’ genomes with free recombination. Each heritable element, either from the weight vector or the contribution matrix, has an equal probability of coming from either parent.

### Simulation experiments

For all our experiments, we compared populations that started genetically uniform but evolved in different types of environments. For the first set of experiments, some populations evolved with “completely correlated changing optima,” meaning all trait optima changed in synchrony every few generations, in the same direction and with the same magnitude of change. Varying in a correlated manner restricts the optimum movement to the points on the diagonal between all axes. We compared the results of these to other populations that evolved with “independently changing optima.” In this second type of environment, the traits’ optima changed independently. This uncorrelated change in the fitness landscape allows for many more possible combinations of trait optima in the trait space.

Changes to the environment happened every 10 generations (every 10,000 births). The magnitude of the change in the environment was ± 0.2. Environmental values are real numbers, but since changes are restricted to the discrete value of 0.2, the optima will only occupy a subset of all real numbers. Changes to the optima increased or decreased with a probability of 0.5 for either movement. The position of the optima was restricted to the interval [−1,1] in all cases, except in our last experiment, where there was no restriction of the environmental value. When the optima reached a boundary, the optima had a 50% chance of staying at the same position or moving away from the boundary. The optimum is thus expected to spend the same amount of time at any given point of the set of possible points.

For each set of conditions, we ran 500 replicates in the completely correlated changing optima environment and 500 replicates in the independently changing optima. Populations evolved in each condition for five million birth–death events. During the last tenth of the run, we collected data from each replicate population every ten generations. A generation is a complete cycle of 1,000 birth–death events. The data collected were the weight vector and contribution matrix of all individuals, along with the position of the optima at that time point.

We also had a third type of environmental change called block-correlated changing optima. In this type of change, the traits were separated into groups. There was a correlated change in their optima within the groups, but the traits in different groups had independent changes of their optima. Similar to the previous experiments, we ran simulations for these environments and compared their result with the independently changing optima.

For most of our experiments, we had a constant number of 4 traits and 20 genes. For some of the block-correlated environments, we also ran simulations with eight traits and 72 genes. The probability of mutation per gene in the weight vector was μ_a_ = 1.25 × 10^−3^ per individual birth. The probability of mutation per gene in the contribution matrix was μ_c_ = 3.125 × 10^−4^. These mutation rates account for 5 mutations of *a* and c type in the population per generation.

### Measuring genetic variance and covariance

A classical description of the population variances and covariances of multiple traits is the additive genetic variance–covariance matrix (**G**) (Lande, 1979). The **G**-matrix diagonal represents the additive genetic variances, and the off-diagonal gives the additive genetic covariances. For asexual populations we do not strictly measure **G**, as we do measure the general genetic variance and covariance of the population. For sexual populations, we measure both the general genetic variance and covariance as well as specifically the additive genetic variance and covariance, thus **G**.

The first question was whether there was a difference in the total genetic variance–covariance matrix of populations evolving in different types of changing environments. For each replicate in each population, we calculated the total genetic variance–covariance matrix of the individuals present in each time point that we collected data. Each genetic variance or covariance was then averaged over all measured time points for a given replicate. We compared the variances and covariances in populations with correlated changes to those from independent changing environments with *t*-tests. All comparisons between two experiments throughout this work were performed using the *t*-test.

For the sexual populations, we also measured additive genetic variances and covariances. Note that despite not having environmental effects that could alter the phenotypic variance, there was potential for epistatic effects between the **a** and the **c** genes; therefore, an experiment is necessary to separate additive genetic variances and covariances from the total genetic variance and covariance. For each recorded time point of each replicate population, we matched 1,000 random couples and calculated their phenotypes and the phenotype of their offspring. To measure the average heritability of the traits, we calculated the slope of the linear regression between the midparent phenotype and the mean offspring phenotype. The additive variance of the population at that time point is the heritability times the variance of the midparent phenotypes. For the covariance, on the other hand, we measured the slope of the linear regression between one trait for the midparent and a different trait for the offspring. Additive genetic covariance was the result of the measured slope multiplied by the variance of the trait used for the midparent.

### Linkage disequilibrium and pleiotropy of total genetic variance and covariance

To determine how much of the genetic variance and covariance that we observed was due to linkage disequilibrium and how much was due to pleiotropy, we randomized alleles among individuals within a time point and within a replicate, for each of the 50 time points of each replicate, to remove the effect of linkage disequilibrium. We calculated the genetic variances and covariances of the population based on the shuffled alleles, to measure how much pleiotropy played a role in the difference between the variances and covariances in the two types of environments. Shuffling the genetic values would only affect the nonrandom association between alleles (linkage disequilibrium), but the underlying alleles and their effects on traits would remain unchanged. Therefore, the covariance after shuffling is fully caused by pleiotropy. We calculated the variance and covariance due to linkage disequilibrium by subtracting the shuffled-population values from the full variance or covariance.

### Mutational variances and covariances

We measured whether the genotype–phenotype maps that evolved under different types of changing environments led to differences in mutational variance and covariances. We collected data on each simulated population at 50 different time points after it had adapted to the environmental condition. To measure the mutational variance and covariance in each population, we randomly chose individuals and measure their phenotypes before and after a single mutation. The mutational variance (or covariance) is proportional to the change in variance (or covariance) caused by a single mutation; we will call these changes M^*^_ij_ (which if *i* = *j* gives the change in the variance for trait *i* for a single mutation or change in covariance of traits *i* and *j* if i≠j). The mutational variance–covariance matrix can be found by multiplying M^*^ by the genomic mutation rate. We further refined our analysis by tracking mutations that occurred in the four weight genes (**a**) separately from those that affected the 16 **c** genes. Only **a** genes can be pleiotropic as the model constrains each element of **c** to only possibly affect a single trait. (However, the values of **c** determine the amount of pleiotropy of the **a** genes.) At each of the measured time points, we measured phenotypes before and after mutation of one million randomly sampled individuals. We measure the difference between the original phenotype and the mutated one (called $\Delta z_{i}$ below).

We find the effects of new mutations on the genetic variance vias the change in the variance of phenotypes before *z*_*i*_ and after mutation *z*’_i_:


$$\begin{array}{l} M_{ii}=Var(z_{i}^{\prime })-Var(z_{i})\; \end{array}$$

which can be simplified to:


$$\begin{array}{l} M_{ii}=Var(\text{Δ}z_{i})\;+2Cov(\text{Δ}z_{i},z_{i}) \end{array}$$

where,


$$\begin{array}{l} \Delta z_{i}=z_{i}^{\prime }-z_{i} \end{array}$$

The mutational covariances can be found from:


$$\begin{array}{l} M_{ij}=Cov(z_{i}^{\prime },z_{j}^{\prime })-Cov(z_{i},z_{j})\; \end{array}$$

which can be calculated as


$$\begin{array}{l} M_{ij}=Cov(\text{Δ}z_{i},\text{Δ}z_{j})+Cov(z_{i},\text{Δ}z_{j})\;+Cov(\text{Δ}z_{i},z_{j})\; \end{array}$$

Each of these can be written with a subscript *a* or *c* to indicate effects from mutations of the *a* genes or *c* genes.

The total mutational matrix was calculated using the mutation rates for the *a* and *c* genes, weighted by the number of *a* and *c* genes (4 and 16, respectively) in the model:


$$\begin{array}{l} M_{tot}=4\mu_{a}M_{a}+16\mu_{c}M_{c} \end{array}$$

### Fitness consequences of the evolution of genetic covariance

We wanted to determine whether the patterns of pleiotropy that evolve in different environmental patterns affect fitness. To do so, after the 5,000 generations of evolution described above, we fixed all individuals in each replicate population for the most common values of the contribution matrix from that replicate to stop future evolution of pleiotropy in those populations. We then copied each replicate population three times and exposed one copy of each to each environmental pattern (correlated, independent, block-correlated changes) for a further 2,000 generations. In these new conditions, each replicate population’s contribution matrix was fixed without new mutations, but the variability and the mutation in the weight vectors continued as during the main simulations.

In these fitness experiments, we removed the limits for the position of the optima in the new environments so that the optima could be any real number. In total, we had nine resulting sets of 500 populations (three types of ancestral conditions times three test conditions). We measured the mean fitness of each replicate every ten generations. After 2,000 generations, we compared the mean fitness of the sets of 500 replicate populations, using the average for the last 10% of the run for each replicate.

## Results

### Evolution of the genetic variance–covariance matrix

To understand the effects of environmental change on the genetic variances and covariances, we compared the simulated populations evolving in different types of changing conditions. Our first comparison asked if correlation of the changes in selective optima would lead to the emergence of positive covariance of the traits selected in those environments. We compared populations evolving in environments in which the selective optima of different traits changed in correlated way with populations that were exposed to independently changing selective pressures.

**Figure 1.**
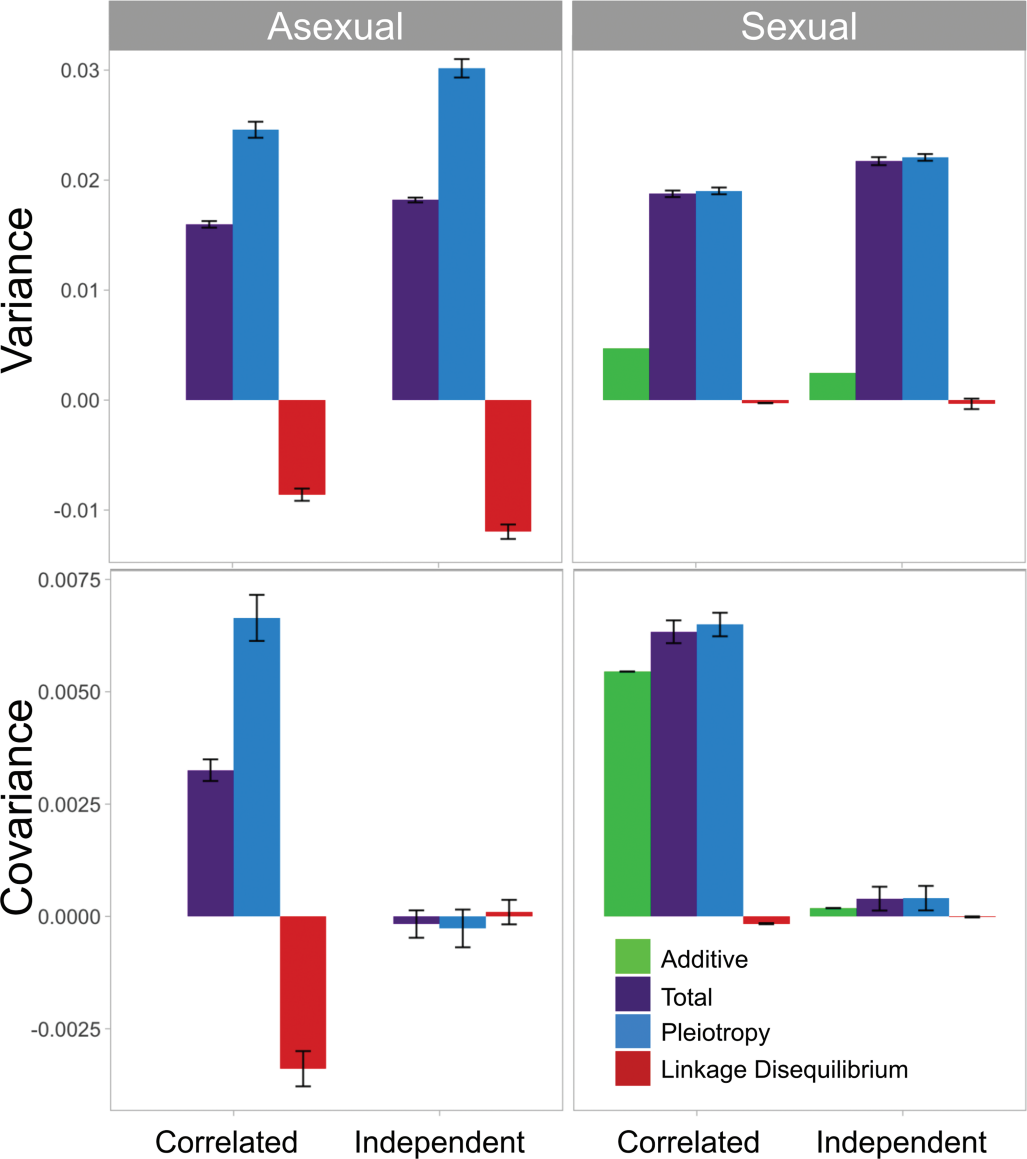
(Top) Comparison of variance values between populations exposed to correlated and independent environments for asexual and sexual populations. The different coloured bars show the decomposition of variance in total variance (purple), variance caused by pleiotropy (blue), variance caused by linkage disequilibrium (red), and additive genetic variance (green). In asexual and sexual populations independent environments led to higher variance. Most of the variance is caused by pleiotropy in all four scenarios. (Bottom) Comparison of covariance values between populations exposed to correlated and independent environments for asexual and sexual populations. Populations evolved in correlated environments had higher covariances, and most of the covariance was explained by pleiotropy. Error bars describe the 95% confidence interval using the average over time of all 500 replicates.

Genetic variances were significantly different between populations that evolved in correlated environments and those evolving in independently changing environments, for both asexual (*p* < 2.2 × 10^−16^; note that this is the default lower limit of *p*-values in R) and sexual populations (*p* < 2.2 × 10^−16^). On average, independently changing selective pressures led to slightly higher genetic variances compared to the populations with correlated changes, around 1.14 times larger for asexual populations and 1.12 for sexual populations (see Supplementary Table 1 and Figure 1, top panels). On the other hand, the differences between genetic covariances were more pronounced (Figure 1, bottom panels). In asexual populations, those that evolved in correlated environments had around 19.2 times higher and more positive covariance values compared to independently changing environments (*p *< 2.2 × 10^−16^). For sexual populations, the total genetic covariance in correlated environments was 16 times larger than in independently changing environments (*p* < 2.2 × 10^−16^). In sexual populations, the additive genetic covariance was on average 40 times higher in the correlated environment populations relative to the independently changing environment populations (*p* < 2. 2 × 10^−16^). Additive variance was on average twice as large in correlated populations (*p* < 2. 2 × 10^−16^). Therefore, the pattern of environmental change had a strong effect on the genetic correlation of these traits.

### Decomposition of the variance–covariance matrix

There are two potential sources of genetic covariance: pleiotropy and linkage disequilibrium. To discover the amount to which each of these sources contributed to the total variance and covariance, we remeasured the variance–covariance matrix after shuffling the alleles among all 1,000 individuals for each collected time point for all 500 populations. The shuffling of alleles removes the effect of linkage disequilibrium; the remaining variance and covariance is due to pleiotropy. The difference between this value after shuffling and the original measures the amount of variance or covariance due to linkage disequilibrium.

In asexual populations, we observed that pleiotropy is responsible for the largest, positive fraction of both variance and covariance in populations that evolved in correlated environments (Figure 1). Linkage disequilibrium decreased both genetic variance and covariance, but the effect was much smaller than pleiotropy in magnitude. The variance (*p* < 2.2 × 10^−16^) and covariance (*p* < 2.2 × 10^−16^) due only to pleiotropy was strongly different between correlated changing environment populations and independently changing environment populations. We also observe a significant difference in variance (*p* = 3.267 × 10^−14^) and covariance (*p* < 2.2 × 10^−16^) caused by linkage disequilibrium alone when comparing the two environment types. When comparing covariance and variance of our correlated changing environment populations before and after swapping we observe a significant difference in both results (variance: *p* < 2.2 × 10^−16^, covariance: *p* < 2.2 × 10^−16^); that is, there is a statistically significant amount of genetic covariance caused by pleiotropy in the populations that evolved with correlated environment change.

Our results were similar for sexual populations. The main difference between the results for asexual and sexual populations was that there was no significant effect on variance from linkage disequilibrium in the sexual populations (*p* = .17). There was still a significant difference in covariance due to linkage disequilibrium, which was 14.7 times larger in correlated populations than in independent populations (*p* < 2.2 × 10^−16^). Similar to the asexual populations, there was a large and significant difference between the variance (*p* < 2.2 × 10^−16^) and covariance (*p* < 2.2 × 10^−16^) due to pleiotropy, in correlated changing environment populations versus independently changing environment populations.

### Mutational variance and covariance

We compared mutational variances and covariances of populations exposed to correlated and independently changing environments in asexual populations. We measured mutational variance and covariance for the **a**-vector portion of the genome, for the **c**-matrix portion, and the total mutational variance and covariance.

Results are presented in Table 1. The ***a*** vector portion of the mutational variance in the populations of independent environments is significantly (*p* < 2.2 × 10^−16^) larger than correlated environments. The ***a*** vector portion of the mutational covariance is significantly different (*p* = 1.5 × 10^−12^) between the two environments, with correlated populations having higher and more positive mutational covariance. There was no significant difference in the ***c****-*matrix portion of mutational variance or covariance between correlated and independent environments. Finally, the total mutational variance was significantly different (*p *< 2.2 × 10^−16^) between both treatments, as well as the total mutational covariance (*p *= 1.5 × 10^−12^). Similar results were observed in simulations with larger numbers of traits or more blocks (Supplementary Figure S1).

**Table 1.** Mutational variance and covariance of populations that evolved in the correlated or independently changing environments. The 95% confidence interval was calculated using the average over time of all 500 replicates.

	Mutational variance		Mutational covariance	
Environment	Correlated	Independent	Correlated	Independent
***a*** vector mutations	2.0 × 10^−6^ ± 8.5 × 10^−8^	2.6 × 10^−6^ ± 9.9 × 10^−8^	2.1 × 10^−7^ ± 4.7 × 10^−8^	6.2 × 10^−8^ ± 5.8 × 10^−8^
*p* value	<2.2 × 10^−16^		1.5 × 10^−12^	
***c***-matrix mutations	9.2 × 10^−12^ ± 3.9 × 10^−12^	1.3 × 10^−11^ ± 4.5 × 10^−12^	−1.4 × 10^−12^ ± 2.0 × 10^−12^	7.0 × 10^−13^ ± 2.7 × 10^−12^
*p-*value	0.19		0.21	
Total mutational variance/covariance	1.0 × 10^−8^ ± 4.2 × 10^−9^	1.3 × 10^−7^ ± 4.9 × 10^−9^	1.0 × 10^−8^ ± 2.4 × 10^−9^	−3.1 × 10^−9^ ± 2.9 × 10^−9^
*p-*value	<2.2 × 10^−16^		1.5 × 10^−12^	

### Emergence of modularity

We further extended our study of the effects of environmental change on genetic variances and covariances by creating a more complex type of change that involved the coupling of sets of selective pressures, which we refer to as blocks. In the block-correlated condition, selective optima changed in a correlated way within each block of traits but independently between blocks. We compared populations evolving in the block-correlated environments with populations evolving in the independently changing condition. We also compared the results within and between blocks. These analyses were performed only in asexual populations.

**Figure 2.**
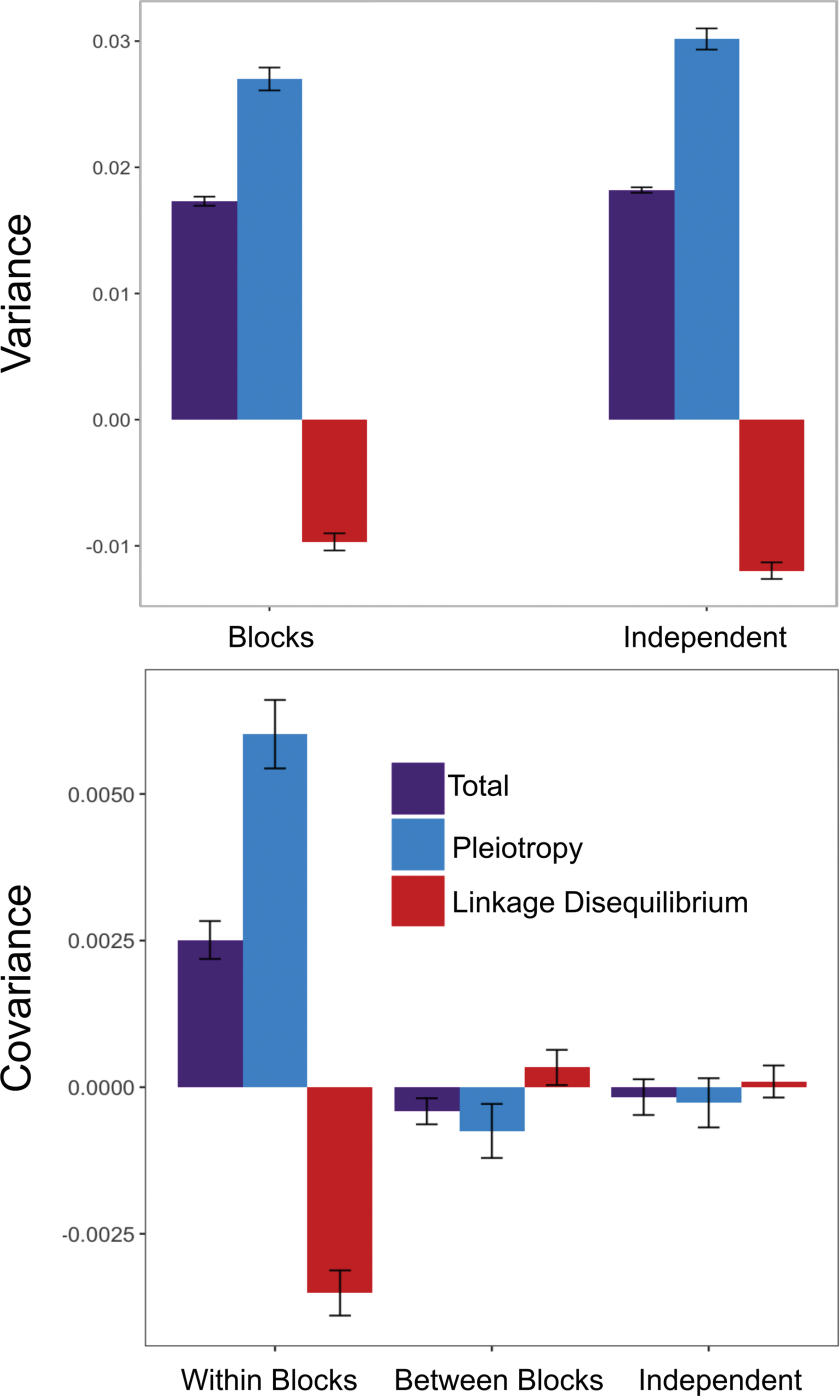
(Top) Comparison of the decomposition for the variance of asexual populations exposed to block-correlated and independent environments. There was no significant difference in variance between the two experiments. (Bottom) Comparison of the decomposition for the covariance within blocks, between blocks, and independent populations. Within blocks refers to the sets of traits whose selective pressures vary in a correlated pattern. Between blocks refers to the sets of traits whose selective pressures vary in an independent pattern. The covariance within blocks was greater than the covariance between blocks. Error bars describe the 95% confidence interval using the average over time of all 500 replicates.

There was a significant (*p* = .00025) difference in genetic variance between populations that evolved in the independently changing environments and populations that evolved in the block-correlated environments. The average genetic covariance of the correlated blocks of traits in the populations that evolved in the block-correlated condition was 15 times higher and more positive than the covariance in the populations that evolved in the independently changing condition (*p* < 2.2 × 10^−16^). Moreover, genetic covariance was 6.1 times higher for traits within the same blocks than for traits in different blocks (*p* < 2.2 × 10^−16^) (Figure 2). (Note that the denominator of these comparisons is a very small number where the standard error is large relative to the magnitude, so these ratios of covariances are noisy.) There was no significant difference when comparing the covariance of different blocks in the block-correlated environments with the covariance of the independently changing environments (*p* = .05711).

We also performed a decomposition of the variance–covariance matrix for these results. We removed linkage disequilibrium by shuffling the alleles among individuals for each time point of each replicate. Different from the comparisons using total variance, we observe a significant difference in variance due to pleiotropy alone between populations evolved in the block-correlated environment and those with independently changing optima (*p* = 5.9 × 10^−16^). Genetic covariances due to pleiotropy were also significantly different between the two conditions when comparing traits within correlated blocks with the results for populations that evolved in the independently changing optima, 22.5 times higher and more positive for within blocks (*p* < 2.2 × 10^−16^). The difference was also significant (*p* < 2.2 × 10^−16^) and even higher, 8 times, when comparing the pleiotropy portion of covariance. There was still no significant difference when comparing between blocks and the independently changing environments (*p* = 0.063).

For the linkage disequilibrium component of the genetic variance, we observed a significant difference between the two conditions (*p* = 2.5 × 10^−16^). The difference was also highly significant (*p* < 2.2 × 10^−16^) when comparing the covariance of within blocks and independently changing environments. There was also a significant difference when comparing the covariance of within blocks and between blocks (*p* < 2.2 × 10^−16^). The linkage disequilibrium portion of the covariance for traits in different blocks was not significantly different from the independently changing case (*p* = .16).

### Evolvability of fitness

**Figure 3.**
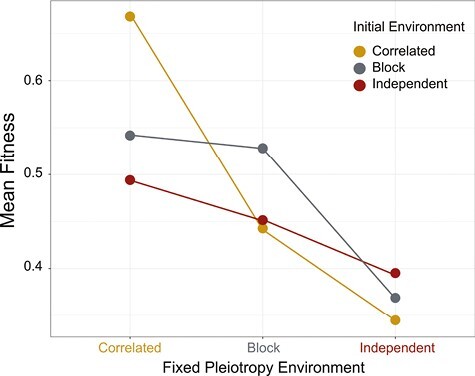
Fitness comparisons between populations evolved in response to the three patterns of environmental change. The results displayed here refer to the experiment in which we fixed the pleiotropy of each population in a set of replicate populations and subsequently exposed them to the three different environmental patterns. The horizontal axis shows the three different conditions that populations with fixed pleiotropy were exposed to after their initial evolution. The different colors of the circles indicate which initial environment the population was exposed to before pleiotropy was fixed. Mustard yellow represents populations initially exposed to correlated environments, blue represents block-correlated environments, and crimson represents independent environments. Populations with pleiotropy evolved in each environment evolve fitness faster in environments with changes in the selective optima that matches their original environment. Means are shown with 95% confidence intervals, but these are so narrow as to be invisible with the size of the points. The confidence interval was calculated using the average over time of all 500 replicates.

After 5,000 generations evolving in a particular environment, we sampled 1,000 individuals from each population and exposed them to the three types of environments we have worked with (correlated, block-correlated, and independent). In general, populations had higher fitness when exposed to the same set of environments that they had evolved in for the first 5,000 generations (Figure 3). Populations that evolved in the correlated environment for the first 5,000 generations and were subsequently exposed to the correlated environment had the highest fitness.

## Discussion

We are still building our understanding of what drives the evolution of traits’ genetic covariance and of the evolutionary consequences of changes in the genetic variance–covariance matrix. It has long been predicted (Lande, 1979) that the response to selective pressure, and consequently adaptive divergence (Schluter, 1996) is predicted by the direction of the largest variance/covariance. Less attention has been given to the possibility that, in fact, not only does genetic covariance affect the patterns of adaptation, but the genetic variance and covariance may be shaped by the evolutionary histories of the population in such a way as to increase genetic variation in the directions that the selective optima are most likely to change. In this article, we demonstrate one way that selection can shape the genetic covariance between traits: the correlation in the changes of selective pressures.

Previous studies have identified at least three selective causes for the evolution of covariance along the directions of divergence among populations or species: plasticity (Draghi & Whitlock, 2012), correlational selection (Sinervo & Svensson, 2002), and migration (Guillaume & Whitlock, 2007). We extend the work of Jones et al. (2004) by proposing that correlated changes in selective pressures can cause adaptive changes in the genetic variance matrix. Through a series of simulations, we demonstrated that selection changing over multiple generations consistently results in genetic covariances that parallel such environmental changes. Our results show that populations exposed to environments that change optima in a positively correlated way evolve positive covariance between the traits affected by those pressures. When lineages are exposed to a set of optima (either continuous or discrete) that are restricted by a particular angle of correlation, the major axis of the genetic covariance follows the same direction as that correlation of the selective optima. Here we demonstrate that this effect can happen through environmental changes over multiple generations and that it does not depend on plasticity.

The evolution we observed in the genetic covariance matrix allowed greater fitness gains in the same environmental regime. Our findings demonstrated that populations increase in fitness fastest when they are exposed to the environmental patterns in which they evolved. Their pleiotropy levels, in particular, are the primary agent of the advantage of populations exposed to the patterns of environmental change into which they evolved.

Expanding on our results, we expect that the patterns of environmental change should affect the expected future direction of adaptive divergence. Our findings show that the axis of greatest genetic differentiation aligns with the direction of covariation in changes in the selective environment, predicting a faster evolutionary response in those directions in the future. Consequently, environmental change may define the path of least resistance, biasing divergence in the direction of previous environmental changes experienced by the population. If future changes in selective optima are correlated with past fluctuations in changes in the optima, populations are more likely to have a rapid response to future changes in the environment than they otherwise would. The implications of oscillating correlated pressures for species diversification should be investigated in future research.

We also explored the genetic causes for the difference in covariance between environments. Pleiotropy is the main reason for the trends in our results. This correlated change in the selective optima causes the evolution of developmental systems to have greater pleiotropy among traits that covary in changes in optima with temporally heterogeneous selection, as evidenced by the changing patterns of effects of new alleles shown in Table 1.

We found that when the pattern of environmental change tended to select for positively correlated change in a pair of traits, that linkage disequilibrium tended to create a negative genetic covariance component for those traits. In our models, traits undergo selection based on a Gaussian fitness function, meaning that the fitness function has a negative curvature for trait means that are relatively close to the new optimum. Such negative curvature in phenotypic selection would translate into negative epistasis at the level of selection on the allele, which predicts an accumulation of negative linkage disequilibrium (Charlesworth, 1993). However, this negative genetic covariance generated by linkage disequilibrium has a lower magnitude than the positive covariation caused by pleiotropy, leaving the traits, on average, positively genetically correlated.

The significant differences in pleiotropy between experiments made us aware of the potential effects of environmental change patterns on the evolution of modularity. We refer to modularity as sets of connected subunits of genes that affect similar traits. Genome-wide studies often show that the relationship between genes and traits occurs modularly. The evolution of modularity has long been debated, and some authors have proposed environmental change as an explanation for genome modularity (Clune et al., 2013; He et al., 2009; Kashtan & Alon, 2005; Kashtan et al., 2007; Lipson et al., 2002; Wagner & Altenberg, 1996; Wagner et al., 2007). Lipson et al.’s (2002) expectation that environmental change could cause the evolution of modularity was supported by simulation experiments (He et al., 2009; Kashtan & Alon, 2005). However, both experiments relied on particular conditions, either nonlinear (and abiological) interactions between traits or horizontal gene transfer. We here show that with reasonable biological assumptions, correlated (and clustered) changes in trait optima can create modular patterns in genetic variation.

We also observe that the patterns of selection on the multiple traits are echoed in the evolution of the mutational variance and covariance of those same traits. In populations in which the selective optima of a pair of traits change in a correlated way, mutational effects evolve to be more correlated among those traits in a way that increases the amount of genetic variation contributed by mutation along the same axes that selection most strongly operates. Such mutational correlations do not evolve in the populations exposed to unrelated changes in their selective optima.

Such changes in the patterns of mutations derive from epistasis in the simple development system that we model here. Genetic covariance can evolve without epistasis if there is variation in the bivariate allelic effects of new mutations (as seen, e.g., in the models of Jones et al., 2004). Such models do not allow the evolution of mutational pleiotropy because the bivariate distribution of phenotypic effects is fixed by assumption. In addition, if alleles at multiple loci interact to create the phenotypes seen by selection, the developmental system itself can evolve in ways that make certain patterns of effects on multiple traits more or less likely. (This shift in the distribution of which phenotypes are easier to create has been called “developmental bias” by Uller et al., 2018.) For example, Draghi and Whitlock (2012) found that the evolution of phenotypic plasticity for multiple traits whose optima covaried created a developmental system that was more likely to create variation in the dimensions of traits space that varied in selective optima over time. Draghi and Whitlock (2012) also found in this model that these changes in the developmental system caused the evolution of mutational pleiotropy. In the current article, we show that heterogeneous and correlated changes in the selective optima over multiple generations changes the simple developmental system to facilitate more pleiotropy. This increase in the pleiotropy of the developmental system is reflected in the higher correlations of mutational effects in the populations from correlated environments compared to the uncorrelated cases. This increase in the genetic covariance along axes of phenotypic space that are under greatest selection may contribute to the patterns observed by Schluter, Houle, and others (Houle et al., 2017; Rohner & Berger, 2023; Schluter, 1996) showing greater genetic variance in directions of divergence among populations or species. Empirical evidence (Estes et al., 2005; McGuigan et al., 2014) shows that mutational pleiotropy is common in different taxa, and here we showed how it can come as a result of selection. Our work contributes to the growing list of phenomena that may cause the evolution of developmental systems and mutational pleiotropy as a function of patterns of selection on the phenotypes over space or time (Jones et al., 2007; Pavlicev et al., 2011; Svensson & Burger, 2019; Svensson, 2022).

The patterns of genetic variance and covariance among traits clearly affect the pace and direction of future evolution, at least in the short term. We have seen that the reverse is sometimes true: that patterns in the change of selective optima can be reflected in the evolution of genetic covariance of relevant traits. Separating cause and effect in studies of the genetic covariance and the direction of evolution will not be simple in natural populations.

## Supplementary Material

qrad048_suppl_Supplementary_Tables_1_Figures_S1

## Data Availability

All scripts necessary to recreate the results described in this manuscript are available on the Github repository: https://github.com/isadoo/doO_Whitlock2023.
