# Supplementary material for: The evolution of genetic covariance and modularity as a result of multigenerational environmental fluctuation
Source: Evol Lett. 2023 Oct 17;7(6):457–66. doi: 10.1093/evlett/qrad048 (PMC10692997; doi:10.1093/evlett/qrad048)
Supplement: qrad048_suppl_Supplementary_Tables_1_Figures_S1 [file qrad048_suppl_supplementary_tables_1_figures_s1.pdf]

Supplementary Materials

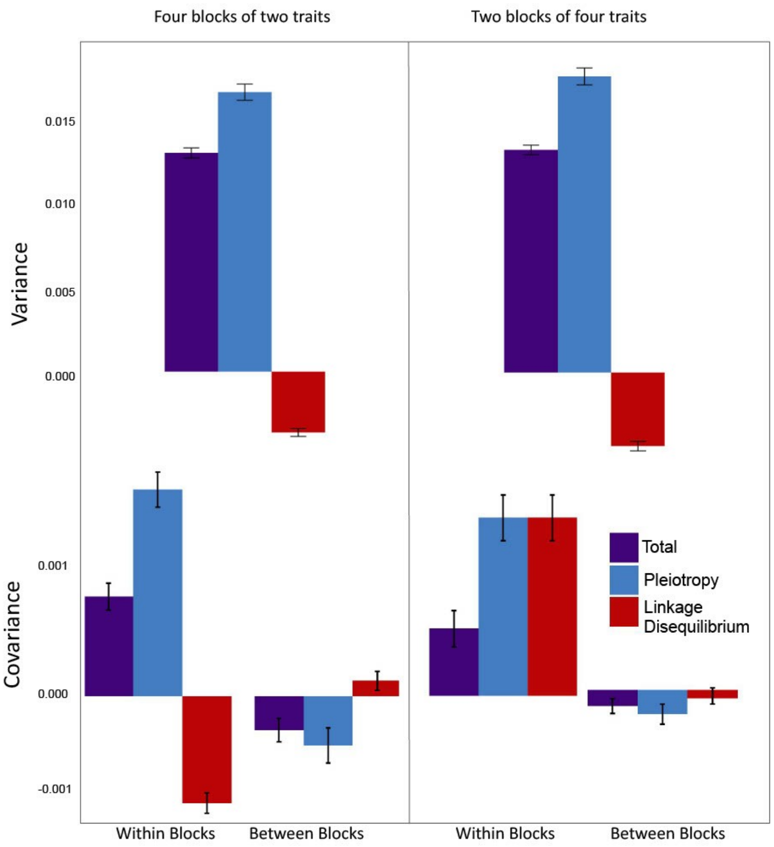

Figure S1. Results for two types of populations exposed to block-correlated environments, with individuals that had eight traits. In both populations individuals were described by an ***a*** vector with eight loci, and a ***c*** matrix with 32 loci. In one case, on the left, populations were exposed to an environment where four pairs of traits had correlated optima while between pairs the change in optima was independent. On the other case, on the right, there were two independent blocks of four traits, in which within the blocks they were affected by correlated changing optima and within the blocks the optima were independent. *Top graphs:* Comparison of variance values. Similar to our previous results, most of the variance is caused by pleiotropy in these new scenarios. *Bottom graphs.* Comparison of covariance values. These results are consistent with the ones described in the main text for the block-correlated populations with four traits.

Table S1. Summary of results on Total Genetic Variance and Covariance

	Total Variance	Total Covariance	Pleiotropy Variance	Pleiotropy Covariance	LD Variance	LD Covariance
Correlated (Asexual)	0.016	0.0032	0.025	0.0066	-0.0086	-0.0034
Independent (Asexual)	0.018	-0.0002	0.03015	-0.0003	-0.012	9.62 x 10^{-05}
Block Correlated Within Blocks (Asexual)	0.01731	0.0025	0.027	0.0060	-0.0097	-0.0035
Block Correlated Between Blocks (Asexual)		-0.0004		-0.00075		0.00033
Correlated (Sexual)	0.019	0.0063	0.019	0.0065	-0.00032	-0.00016
Independent (Sexual)	0.021	0.00040	0.021	0.00041	-0.00030	-1.09
Correlated Additive (Sexual)	0.0047	0.0074	Does not apply.			
Independent Additive (Sexual)	0.0027	0.00019	Does not apply.			
